# Mind Cure Convention

**Published:** 1888-02

**Authors:** 


### MIND CURE CONVENTION.

### DR. BARTOL, WHO WAS EXPECTED TO DEFEND THE THEORY, TOOK THE OTHER SIDE.

In Boston the mind cure enthusiasts were having a two days' convention, and were discussing their peculiar faith with a great deal of fervor. They regarded the Rev. Dr. C. A. Bartol as one of their ablest defenders, and he was announced to speak on "The Pro and Con of Mind Cure." What he said on the con side took the convention all aback. He said: "There is danger of extravagance. The mental healers have not a monopoly. They cannot kill off the old doctors. Can Christian science set a broken limb? It might take the beam out of the eye, but a cinder is too much for you."

Mrs. Diaz interrupted the speaker and asked if he had ever tried the mental healing on a cinder.

Dr. Bartol replied that he tried it, but finally went to a doctor and had the cinder removed. Continuing he said:

"Let us be true; let us be consistent. But you can't put aside all the old theories. As Dr. Bowditch said, I have yet to see the mental healing that can destroy the germs of typhoid fever. Do not suppose that you can in a moment become the highest type of mind-cure or any other kind of physician. I do not believe that a man can come from behind a counter or from an express wagon and the next day be a good doctor. There are specific tonics in medicine that have their virtues, and you cannot do away with them. Do not think that I am on the fence. I am on both sides of it."

Some one rose in the back of the hall and said he would like to ask a question. He was permitted to do so, and he called out: "Is it possible to successfully face both ways?"

Dr. Bartol raised a laugh by answering: "I look all around."

### HER TERRIBLE DREAM CAME TRUE TO THE LETTER.

Mrs. Jacob Condon, living a few miles from this place, dreamed a few nights ago that her year-old baby was burned to death, and that she sent word of the casualty to her husband, who was working at a distance from home, by James Portlewaith, a neighbor. The next morning she told her husband of her dream, and admitted that it made her despondent. He laughed at her fears, and went away to his work. Late in the forenoon Mrs Condon left her kitchen to go to the woodshed, a few steps away. While she was there she heard her baby screaming. She ran into the house, and found the child lying in front of an open grate, wrapped in flames. She threw an old coat about the child and smothered the flames, but it was so badly burned that it died in a few minutes, Mrs. Condon went to the door to call for assistance. As she reached the door James Portlewaith was passing the gate. She sent him to her husband with the dreadful news, thus fulfilling her dream to the letter.

